# The relationship between social support and college students’ physical activity participation in China: the mediating effect of self-efficacy

**DOI:** 10.3389/fpsyg.2025.1596841

**Published:** 2025-06-25

**Authors:** Mingtian Niu, Mingyuan Dong, Panpan Shi, Yongchul Choi, Ning Li

**Affiliations:** ^1^Department of Public Physical Education, Xinyang College, Xinyang, China; ^2^Department of Physical Education, Gangneung–Wonju National University, Gangneung, Republic of Korea; ^3^School of Computer Science, Wuhan University, Wuhan, China

**Keywords:** college students, mediating effect, participation in physical activities, self-efficacy, social support

## Abstract

**Background:**

With the increasing health awareness among Chinese college students, the relationship between social support, self-efficacy, and participation in physical activities has become a focal point of research. Particularly in the context of gender differences, exploring how these factors influence college students’ exercise behaviors is significant.

**Objective:**

This study aims to examine how social support influences college students’ self-efficacy and their participation in physical activities, and whether self-efficacy mediates this relationship. It should be noted that in the context of this study, college students are considered late adolescents or emerging adults, as defined in developmental psychology.

**Methods:**

A survey was conducted among 489 college students from universities in Henan Province, China. The survey included the Physical Activity Questionnaire (PAQ), the Social Support Scale, and the Exercise Self-Efficacy Scale (ESES). Exploratory factor analysis, linear regression analysis, and structural equation modeling were used to examine the relationship between social support and college students’ participation in physical activities.

**Results:**

The regression effects of social support on college students’ participation in physical activities (*F* = 47.898) and self-efficacy (*F* = 224.247) were significant (*p* < 0.01). Self-efficacy also significantly predicted participation in physical activities (*F* = 136.706, *p* < 0.01). Among female students, both the effect of social support (*B* = 0.177, *t* = 2.332*) and self-efficacy (*B* = 0.307, *t* = 5.810**) on participation in physical activities were significant. The mediating effect of self-efficacy accounted for 59.6% of the total effect, while the direct effect accounted for 40.4%.

**Conclusion:**

To promote female college students’ participation in physical activities, particular attention should be paid to the critical role of self-efficacy. At the same time, it is also important not to overlook the competitive sports preferred by male students, which require stronger self-efficacy to cope with competitive pressures and social expectations.

## 1 Introduction

The World Health Organization defines physical activity as any bodily movement produced by skeletal muscles that results in energy expenditure, including activities of daily living such as walking, climbing stairs, doing household chores, and engaging in exercise (World Health Organization [WHO], 2010). Recent research highlights its benefits: enhancing cardiovascular health, improving muscle strength, flexibility, aiding in weight management, and reducing risks of chronic diseases (Strain et al., 2024). Additionally, regular physical activity enhances mental health, self-confidence, and self-efficacy (Trajković et al., 2023), while fostering social adaptability, relationships, and a sense of belonging (Carrete-Marín, 2024). Good social adaptability helps individuals integrate and develop better in society, reducing feelings of loneliness and social isolation. However, despite these advantages, a large proportion of the global population remains insufficiently active. According to the Global Physical Activity Report released by the WHO, over 80% of adolescents and 27% of adults do not meet the recommended levels of physical activity (World Health Organization [WHO], 2022). College students—positioned at the transitional stage between adolescence and young adulthood—also show low participation rates, largely due to academic pressures, changing lifestyles, and competing priorities (Guerriero et al., 2025). Recent data from the Report on the Physical Fitness of Chinese Youth indicate a declining trend in the overall physical fitness levels among Chinese college students. Concurrently, it is estimated that 40–50% of college students worldwide do not engage in sufficient physical activity, with key contributing factors including time constraints, social influences, and evolving personal priorities (Pan et al., 2022; Brown et al., 2024).

Studies have confirmed that social support can significantly enhance individuals’ willingness and persistence in participating in physical activities (Schmitke, 2024). Support from family, friends, and coaches can boost exercisers’ confidence and motivation (Mira et al., 2023). Additionally, social support can reduce stress and anxiety during exercise, thereby promoting overall physical and mental health (Lin et al., 2024). By offering positive feedback and encouragement, social support helps exercisers overcome challenges and achieve their goals, playing a critical role in increasing participation rates and maintaining long-term exercise habits. Social support encompasses the various forms of assistance and reassurance individuals receive from their social networks to cope with physiological, psychological, and social stressors. These networks—comprising family, friends, neighbors, religious groups, colleagues, and peer support communities—provide practical assistance (e.g., help with daily tasks, advice), material resources (e.g., financial aid), and emotional support (e.g., fostering feelings of being valued and understood) (Cohen and Wills, 1985). Empirical studies have shown that such support significantly promotes physical activity participation among college students (Zhang et al., 2022). Furthermore, social support influences exercise behavior not only directly but also indirectly by enhancing individuals’ self-efficacy (Brown et al., 2024). Accordingly, the hypothesis was formulated: H1: Social support can significantly improve college students’ participation in physical activities.

The relationship between social support and self-efficacy is closely intertwined. Social support, including encouragement and assistance from family, friends, and colleagues, can significantly enhance individuals’ self-efficacy (Cutrona and Russell, 1990). When individuals perceive support and affirmation from others, they are more likely to believe in their ability to overcome difficulties and achieve goals, thereby strengthening their self-efficacy (Lent et al., 1986). In 1986, Canadian psychologist Albert Bandura defined self-efficacy as an individual’s confidence and belief in their ability to complete specific tasks or behaviors (Bandura, 1986). Accordingly, Hypothesis H2: Social support can significantly improve college students’ self-efficacy.

The importance of self-efficacy lies in its profound impact on an individual’s motivation, self-regulation, and ability to cope with challenges (Bandura and National Inst of Mental Health, 1986). Individuals with high self-efficacy are more likely to set higher goals, persist longer, and demonstrate greater resilience in the face of difficulties. Additionally, self-efficacy can enhance decision-making and problem-solving abilities, reduce stress and anxiety, and thereby promote personal success and growth (Pajares, 1996). Accordingly, hypotheses H3: Self-efficacy can significantly improve college students’ participation in physical activities. Finally, based on the above hypotheses, hypothesis H4: Social support indirectly improves college students’ participation in physical activities through the mediating role of self-efficacy. This hypothesis aims to reveal the mechanism by which social support enhances self-efficacy, thereby promoting physical activity participation.

In summary, college students are in a critical stage of developing social adaptability. Investigating the factors that influence their participation in physical activity during this period is essential for identifying underlying challenges in their academic and personal lives and for designing targeted interventions. Such efforts are fundamental to supporting their physical and mental well-being and fostering the development of a socially integrated identity. The primary aim of this study is to examine the relationships among social support, self-efficacy, and physical activity, with a particular focus on the mediating role of self-efficacy in the link between social support and physical activity participation. By analyzing the interactions among these variables, the study seeks to offer new theoretical insights into the mechanisms underlying college students’ engagement in physical activity and to provide empirical evidence to inform the development of effective intervention strategies.

## 2 Methodology

### 2.1 Participants

This study conducted an online cross-sectional survey in May 2024 using the Wenjuanxing platform. Electronic questionnaires were distributed through university student class groups using convenience sampling. A total of 491 responses were collected, and after excluding incomplete or invalid questionnaires, 489 valid responses were retained, resulting in an effective response rate of 99.6%.

The sample comprised 489 undergraduate students from a private university in Henan Province, with an average age of 19.11 ± 1.034 years. The gender distribution was approximately balanced. The primary objective of this study was to examine the relationships among social support, self-efficacy, and physical participation among college students. Specifically, the study assesses the direct effects of social support and self-efficacy on physical participation, explores the mediating role of self-efficacy, and investigates the moderating effect of gender within the mediation model.

The sample size was determined based on guidelines for structural equation modeling (SEM), which recommend a minimum ratio of 10–20 participants per estimated parameter. Given the model complexity and number of observed variables, a minimum of approximately 300 participants was considered sufficient. Therefore, the final sample of 489 exceeds the recommended threshold, ensuring adequate statistical power. All statistical analyses were conducted using a significance level of *p* < 0.05.

### 2.2 Outcome measures

#### 2.2.1 Questionnaire survey method

##### 2.2.1.1 Questionnaire design

Physical Activity Participation: The Physical Activity Questionnaire (PAQ), developed by Canadian scholars specifically for adolescents, was employed as an assessment tool for physical activity (Kowalski et al., 2004). The most representative component of the PAQ was selected, which includes 7 questions recalling physical activity participation over the past week. Additionally, the Physical Exercise Rating Scale, revised by Chinese scholar Liang Deqing in 1994, was incorporated. This scale consists of 3 questions primarily designed to measure exercise intensity, duration, and frequency (Liang, 1994). The physical activity participation scale comprised a total of 10 questions, scored using a 5-point Likert scale, with higher scores indicating greater levels of participation.

##### 2.2.1.2 Questionnaire details

(1) Social support: the social support scale developed by Sarason et al. (1983) was adopted, which is divided into three dimensions: family, friends, and others. Questions related to peers and family were categorized into two aspects: emotional support (e.g., “When you experience negative emotions during exercise, your friends comfort you”) and behavioral modeling (e.g., “Your parents exercise regularly, and most of your friends also exercise frequently”). School support was further divided into three aspects: academic support (e.g., encouragement from teachers), quality of sports facilities (e.g., “The school has well-equipped sports venues”), and sports culture atmosphere (e.g., “The school frequently organizes large-scale sports events with high participation rates”). The scale and questionnaire items are presented in Table 1, comprising a total of 13 questions. Responses were scored on a 5-point scale, with higher values indicating greater levels of support.

**Table 1:** Differential analysis of variables by gender among college students (*N* = 489).

	Male	Female	T
Physical activity participation	2.9 ± 0.78	2.68 ± 0.653	3.27**
Physical activity participation in the past week	2.89 ± 0.832	2.7 ± 0.733	2.63*
Exercise frequency, intensity, and duration	2.92 ± 0.859	2.62 ± 0.731	4.02**
Social support	3.49 ± 0.647	3.58 ± 0.548	−1.68
Friend support	3.31 ± 0.848	3.38 ± 0.664	−1.01
School support	3.66 ± 0.688	3.78 ± 0.591	−2.13*
Family support	3.46 ± 0.705	3.53 ± 0.658	−1.01
Self-efficacy	3.41 ± 0.784	3.27 ± 0.787	1.82
Physical factor	3.39 ± 0.858	3.18 ± 0.841	2.73*
Psychological factor	3.54 ± 0.832	3.4 ± 0.835	1.78
Physical environmental factor	3.39 ± 0.801	3.24 ± 0.847	1.92
Social environmental factors	3.31 ± 0.873	3.25 ± 0.842	0.84

**p < 0.01, *p < 0.05, significant correlation.

(2) Self-efficacy: the Exercise Self-Efficacy Scale (ESES) was used to assess exercise self-efficacy. The Chinese version of the ESES consists of 18 questions, divided into four domains: physical factors, psychological factors, physical environment factors, and social environment factors (Tung et al., 2005). The classification is primarily based on factors influencing individuals’ confidence in exercising under different circumstances. Specifically, these factors can be categorized as follows:

- Physical factors: These questions focus on individuals’ confidence in exercising when their physical condition changes, such as when they are fatigued, injured, or ill.
- Psychological factors: These questions focus on individuals’ confidence in exercising when their psychological or emotional state changes, such as when they feel depressed, anxious, or face personal issues.
- Physical environment factors: These questions focus on the impact of external environments on individuals’ confidence in exercising, such as bad weather or during holidays.
- Social environment factors: These questions focus on the influence of social and family environments on individuals’ confidence in exercising, such as when they lack support from family or friends or have visitors.

##### 2.2.1.3 Reliability and validity testing of the questionnaire

Principal component analysis was employed to analyze the data for each scale. The Kaiser–Meyer–Olkin (KMO) measure and Bartlett’s test of sphericity were used to assess the structural validity of the questionnaire. As shown in Table 2, all KMO values were greater than 0.8, indicating that the data were suitable for factor analysis. Additionally, Bartlett’s test of sphericity yielded significance levels below 0.001, confirming significant correlations among the variables.

**Table 2:** Validity test of the scales.

		Physical activity participation	Social support	Sports self-efficacy
KMO value		0.860	0.917	0.965
Bartlett’s Test of Sphericity	*χ* ^2^	1640.842	3433.112	8402.013
	df	45	78	153
	Sig.	0.000	0.000	0.000

*χ*^2^, Chi-Square Value; df, degrees of freedom; Sig., significance.

Cronbach’s alpha coefficient was used to evaluate internal consistency reliability. For the physical activity participation scale, the common factor variance ranged from 0.546 to 0.914. The Cronbach’s alpha coefficient for the 10 items was 0.844, with subscales for exercise behavior (0.609) and past-week PAQ (0.819) both exceeding 0.6, indicating strong internal consistency among the items. For the social support scale, the common factor variance ranged from 0.508 to 0.671. The Cronbach’s alpha coefficient for the 13 items was 0.913, with subscales for friend support (0.838), school support (0.872), and family support (0.814) all exceeding 0.8, demonstrating high internal consistency for both the overall scale and its dimensions. For the exercise self-efficacy scale, the common factor variance ranged from 0.742 to 0.849. The Cronbach’s alpha coefficient for the 18 items was 0.968, with subscales for physical factors (0.865), psychological factors (0.925), physical environment factors (0.811), and social environment factors (0.932) all exceeding 0.8, indicating high internal consistency for both the overall scale and its dimensions.

### 2.3 Statistical analysis

The results of the questionnaire survey were organized and analyzed using mathematical statistical software such as SPSS 26.0 and Microsoft Excel. Descriptive statistics, exploratory factor analysis, independent samples *t*-test, Pearson correlation analysis, regression analysis, and structural equation modeling (SEM) were employed to statistically analyze the sample data. An independent samples *t*-test was performed to assess gender differences among college students in terms of their scores on the physical activity participation, social support, and self-efficacy scales. Furthermore, Pearson correlation analysis was employed to explore the associations between students’ engagement in physical activities and their levels of social support and self-efficacy. These methods were used to examine the relationships among variables, identify underlying factors, and test the proposed hypotheses, ensuring a comprehensive understanding of the data and the validity of the findings.

## 3 Results

### 3.1 Analysis of basic information

Through descriptive statistical analysis (Table 3), the mean age of the college students participating in the survey was 19.11 ± 1.034 years. Among them, there were 192 male students and 297 female students. The mean score for participation in physical activities was 2.76 ± 0.713. The mean score for social support was 3.55 ± 0.589, with teacher support (3.73 ± 0.634) being higher than family support and friend support. The mean score for self-efficacy was 3.33 ± 0.788, with psychological factors (3.46 ± 0.835) scoring higher than physical environmental factors, social environmental factors, and physical factors.

**Table 3:** Overview of basic information (*N* = 489).

	Mean/percentage
Age	19.11 ± 1.034
Sex	
Male (192)	39.3%
Female (297)	60.7%
Physical activity participation	2.76 ± 0.713
Physical activity participation in the past week	2.77 ± 0.778
Exercise frequency, intensity, and duration	2.74 ± 0.797
Social support	3.55 ± 0.589
Friend support	3.35 ± 0.741
School support	3.73 ± 0.634
Family support	3.50 ± 0.677
Self-efficacy	3.33 ± 0.788
Physical factor	3.26 ± 0.853
Psychological factor	3.46 ± 0.835
Physical environmental factor	3.30 ± 0.831
Social environmental factors	3.27 ± 0.854

Physical Activity Participation: includes “Participation in Physical Activity in the Past Week” and “Exercise frequency, intensity, and duration”; Social Support: includes “Friend Support,” “School Support,” and “Family Support”; Self-efficacy: includes “Physical Factors,” “Psychological Factors,” “Physical Environmental Factors,” and “Social Environmental Factors”.

An independent samples t-test was conducted on the participation in physical activities, social support, and self-efficacy scales among college students of different genders (Table 1). The results revealed significant differences in participation in physical activities (3.27**), with male students showing significantly higher participation than female students. The differences in social support and self-efficacy were relatively small, but there was a noticeable difference in teacher support within social support (−2.13*) and a significant difference in the physical factors of self-efficacy (2.73*).

Pearson’s bivariate two-tailed correlation test (Table 4) indicated that college students’ participation in physical activities was significantly positively correlated with all indicators of social support and self-efficacy (*p* < 0.01). Among these, friend support within social support (*r* = 0.309) and psychological factors within self-efficacy (*r* = 0.469) showed relatively higher positive correlations.

**Table 4:** Summary of Pearson correlation analysis (*N* = 489).

Variable	PSA	Fr-S	SS	Fa-S	Ph-F	Ps-F	PEF	SEF
PSA	1							
Fr-S	0.309**	1						
SS	0.208**	0.593**	1					
Fa-S	0.265**	0.643**	0.648**	1				
Ph-F	0.409**	0.470**	0.330**	0.528**	1			
Ps-F	0.469**	0.507**	0.409**	0.541**	0.834**	1		
PEF	0.425**	0.493**	0.354**	0.570**	0.826**	0.817**	1	
SEF	0.434**	0.460**	0.332**	0.530**	0.800**	0.809**	0.865**	1

**p < 0.01, significant correlation. PSA, Participation in sports activities; Fr-S, Friend support; SS, School support; Fa-S, Family support; Ph-F, Physical factor; Ps-F, Psychological factor; PEF, Physical environmental factor; SEF, Social environmental factors.

### 3.2 Regression analysis of variables

Using social support as the independent variable and physical activity participation level as the dependent variable, a forced-entry regression analysis was conducted (Table 5). The results showed that social support had a significant regression effect on physical activity participation level (*F* = 47.898, *p* < 0.01), explaining 8.8% of the variance. In the gender-stratified regression analysis, female students required higher levels of social support (0.438) compared to male students, with social support explaining 13.2% of the variance among females. Therefore, Hypothesis 1 (social support can improve college students’ physical activity participation) was validated, and the regression equations for both male and female students were statistically significant.

**Table 5:** Regression analysis of social support on college students’ physical activity participation (*N* = 489).

Variable	Participation in sports activities			Male			Female		
	B	SE	β	B	SE	β	B	SE	β
Social Support	0.362	0.299	0.052**	0.312	0.258	0.085**	0.438	0.367	0.065**
*R* ^2^	0.088			0.062			0.132		
F	47.898**			13.592**			45.977**		

**p < 0.01, significant correlation. *R*^2^ (*R*-squared): proportion of variance in the dependent variable explained by the model; F (F-statistic): Tests overall significance of the regression model; B (Unstandardized Coefficient): Change in the dependent variable for a one-unit change in the predictor; SE (Standard Error): Precision of the coefficient estimate; β (Standardized Coefficient/Beta): Standardized coefficient, showing the effect in standard deviation units.

Using social support as the independent variable and self-efficacy as the dependent variable, a forced-entry regression analysis was conducted (Table 6). The results showed that social support had a significant regression effect on self-efficacy (*F* = 224.247, *p* < 0.01), explaining 31.4% of the variance. In the gender-stratified regression analysis, female students required higher levels of self-efficacy (0.850) compared to male students, with social support explaining 34.7% of the variance among females. Therefore, Hypothesis 2 (social support can improve college students’ self-efficacy) was validated, and the regression equations for both male and female students were statistically significant.

**Table 6:** Regression analysis of social support and self-efficacy (*N* = 489).

Variable	Self-efficacy			Male			Female		
	*B*	SE	β	*B*	SE	β	*B*	SE	β
Social Support	0.751	0.562	0.050**	0.667	0.550	0.074**	0.850	0.591	0.068**
*R* ^2^	0.314			0.299			0.347		
*F*	224.247**			82.390**			158.357**		

**p < 0.01, significant correlation. *R*^2^ (*R*-squared): Proportion of variance in the dependent variable explained by the model; F (F-statistic): Tests overall significance of the regression model; B (Unstandardized Coefficient): Change in the dependent variable for a one-unit change in the predictor; SE (Standard Error): Precision of the coefficient estimate; β (Standardized Coefficient/Beta): Standardized coefficient, showing the effect in standard deviation units.

Finally, using self-efficacy as the independent variable and college students’ physical activity participation as the dependent variable, the impact of self-efficacy on physical activity participation was tested (Table 7). The results showed that self-efficacy had a significant regression effect on physical activity participation level (*F* = 136.706, *p* < 0.01), explaining 21.8% of the variance. In the gender-stratified regression analysis, male students required higher levels of self-efficacy (0.471) compared to female students, with self-efficacy explaining 22% of the variance among males. Therefore, Hypothesis 3 (self-efficacy can improve college students’ physical activity participation) was validated, and the regression equations for both male and female students were statistically significant.

**Table 7:** Regression analysis of self-efficacy and college students’ physical activity participation (*N* = 489).

Variable	Physical activity participation			Male			Female		
	B	SE	β	B	SE	β	B	SE	β
Self-Efficacy	0.424	0.468	0.036**	0.471	0.473	0.064**	0.380	0.458	0.043**
R^2^	0.218			0.220			0.207		
F	136.706**			54.802**			78.226**		

**p < 0.01, significant correlation. *R*^2^ (*R*-squared): Proportion of variance in the dependent variable explained by the model; F (F-statistic): Tests overall significance of the regression model; B (Unstandardized Coefficient): Change in the dependent variable for a one-unit change in the predictor; SE (Standard Error): Precision of the coefficient estimate; β (Standardized Coefficient/Beta): Standardized coefficient, showing the effect in standard deviation units.

### 3.3 Mediation effect analysis of variables

The mediation effect analysis reveals (Table 8) that only the effect of self-efficacy is statistically significant (*B* = 0.397, *t* = 9.061**). However, in the gender-specific regression analysis, it is found that in the female subgroup, both social support (*B* = 0.177, *t* = 2.332*) and self-efficacy (*B* = 0.307, *t* = 5.810**) have significant effects. The regression model *F* = 42.419** and *R*^2^ = 0.219 confirm that the explanatory power of the model is statistically significant. Therefore, the mediation effect of “social support – self-efficacy – participation level of college students in physical activities” is significant. Thus, research hypothesis 4 (social support can enhance college students’ participation in physical activities through the mediating effect of self-efficacy) is validated, particularly significant among female college students.

**Table 8:** Mediation effect test (*N* = 489).

Regression equation		Coefficient				Goodness-of-fit measures	
Grouping variable		*B*	*β*	SE	*t*	*R* ^2^	*F*
Total	Social support	0.064	0.059	0.053	1.098	0.218	68.984**
	Self-efficacy	0.397	0.044	0.438	9.061**		
Male	Social support	−0.003	0.093	−0.003	−0.034	0.216	27.258**
	Self-efficacy	0.472	0.076	0.475	6.185**		
Female	Social support	0.177	0.076	0.148	2.332*	0.219	42.419**
	Self-efficacy	0.307	0.053	0.37	5.810**		

**p < 0.01, significant correlation. *R*^2^ (*R*-squared): Proportion of variance in the dependent variable explained by the model; F (F-statistic): Tests overall significance of the regression model; B (Unstandardized Coefficient): Change in the dependent variable for a one-unit change in the predictor; SE (Standard Error): Precision of the coefficient estimate; β (Standardized Coefficient/Beta): Standardized coefficient, showing the effect in standard deviation units.

Using Model 4 in SPSS as developed by Hayes (2013), with female college students as the sample size, the mediation effect path and effect values (Table 9) indicate that in the study of the relationship between social support and participation in physical activities, the mediation effect of self-efficacy is significant, accounting for 59.6% of the total effect, while the direct effect accounts for 40.4%.

**Table 9:** Mediation effect pathways and effect sizes (*N* = 297).

Effect	Effect	BootSE	BootLLCI	BootULCI	Proportion of effect
Mediation effect	0.261	0.053	0.158	0.367	59.6%
Direct effect	0.177	0.076	0.026	0.327	40.4%
Total effect	0.438	0.065	0.311	0.565	

BootSE, Bootstrap Standard Error; BootLLCI, Bootstrap Lower Limit Confidence Interval; BootULCI, Bootstrap Upper Limit Confidence Interval.

**Figure 1:**
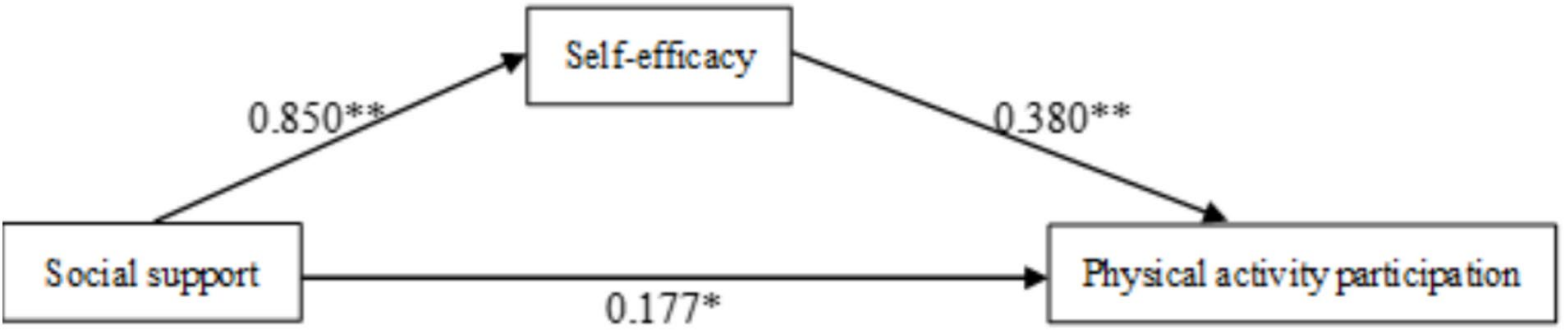
The relationship between social support and physical activity participation: the mediating role of self-efficacy (*N* = 297). **p < 0.01, *p < 0.05, significant correlation.

As shown in Figure 1, social support not only directly affects college students’ participation in physical activities (0.177*), but also enhances their self-efficacy (0.850**). Self-efficacy, in turn, can increase participation in physical activities (0.380**). Moreover, the mediation effect of self-efficacy in the influence of social support on college students’ participation in physical activities is established (0.261**).

## 4 Discussion

### 4.1 Social support as a direct influencing factor of physical activity participation

The regression effect of social support on the level of physical activity participation was significant (*F* = 47.898), explaining 8.8% of the variance. This result indicates that social support is an important factor in promoting individuals’ participation in physical activity. Specifically, when individuals perceive support from family, friends, or school, they are more likely to participate actively. Such support may manifest as encouragement, companionship, or the provision of resources (e.g., sports equipment or venues), thereby reducing barriers to participation and enhancing individuals’ willingness and ability to engage in physical activities (Schmitke, 2024). Social support also enhances motivation, reduces fear of failure, and provides emotional security (Mira et al., 2023). Moreover, it fosters a positive social atmosphere, which strengthens the sense of achievement and belonging during physical activity (Ferreira et al., 2024).

In the context of the Chinese college student population studied, gender differences emerged in this relationship. In the regression equation of social support and physical activity participation levels, it was found that, compared to male students, female students required higher levels of social support (0.438) to achieve the same level of physical activity. There are also significant gender differences in physical activity participation levels, with male students showing significantly higher participation than female students. Moreover, when assessing social support, significant gender differences were observed in school support, with female students receiving more school support than male students, such as through the organization of fun sports events, access to good sports facilities, the promotion of sports culture on campus, and encouragement and support from physical education teachers. This may be related to gender roles and societal expectations prevalent in Chinese society. Traditionally, women are assigned more roles related to emotional expression and interpersonal interaction in society, which makes them more inclined to seek external support when facing pressure or challenges (Chen and Zhang, 2024). At the same time, Chinese women may be more encouraged to rely on others during their upbringing, especially in terms of emotional support, leading to a higher dependence on social support (Johansen et al., 2021). Female students tend to have lower self-efficacy in physical activities, resulting in insufficient confidence to participate (Öztürk et al., 2021). They are also more likely to be troubled by body image and self-esteem issues, which may affect their willingness to engage in physical activities (Sunderji et al., 2024). In some cases, female students have fewer opportunities and resources to participate in physical activities, which further limits their participation levels.

### 4.2 Social support’s role in promoting self-efficacy

The regression effect of social support on self-efficacy was even more significant (*F* = 224.247), explaining 31.4% of the variance. This result indicates that social support plays a crucial role in enhancing individuals’ self-efficacy. Self-efficacy refers to an individual’s belief in their ability to complete a task or cope with a specific situation, and social support can help individuals establish and maintain this belief by providing encouragement, feedback, and role models (Lin et al., 2024). Specifically, when individuals perceive support from others, they are more likely to believe in their own abilities, especially when facing challenges or difficulties. Social support can provide emotional comfort and practical assistance, thereby boosting their confidence and coping abilities (Li et al., 2023). Furthermore, social support can help individuals develop positive self-evaluations through constructive feedback and recognition, further enhancing their self-efficacy (Li and Liu, 2025).

Among Chinese college students, the gender-stratified regression analysis revealed that female students required higher levels of self-efficacy (0.850) compared to male students. This result may be related to gender roles and societal expectations. Additionally, women may face higher societal expectations and pressures in multiple roles, such as academics, careers, and family responsibilities, necessitating stronger self-efficacy to cope with these challenges. The higher demand for self-efficacy reflects the need for women to enhance their self-beliefs to build confidence and motivation when navigating complex social environments.

### 4.3 Self-efficacy as a predictor of physical activity participation

In the context of physical activity participation, students with higher self-efficacy are more likely to engage actively in physical activities because they believe in their ability to complete tasks and cope with potential challenges. This belief can enhance their motivation, reduce fear of failure, and encourage them to try new sports or maintain existing exercise habits. Self-efficacy explains 21.8% of the variance, indicating that it is a significant predictor of Chinese college students’ participation in physical activity. Students with high self-efficacy are generally more confident and persistent, enabling them to maintain a positive attitude when facing difficulties, thereby increasing the likelihood of long-term participation in physical activities (Li et al., 2022). Beyond promoting persistence, self-efficacy also facilitates goal-setting and behavior planning, both of which are essential for lasting physical activity engagement.

In the gender-stratified regression analysis, male students (0.471) required higher levels of self-efficacy. This result may be related to gender roles and societal expectations within Chinese culture. Traditionally, men are assigned more competitive and athletic roles in society, with higher societal expectations for their sports performance (Santisteban et al., 2022). As a result, male students may need stronger self-efficacy to cope with competitive pressures and societal expectations when participating in physical activities. Furthermore, male students may be more inclined to choose competitive or high-intensity sports (e.g., basketball, soccer), which place higher demands on individuals’ physical abilities and psychological resilience (Deaner et al., 2012). Therefore, male students require higher self-efficacy to believe in their ability to excel in these sports and perform well in competitive settings.

### 4.4 Self-efficacy as a mediator between social support and physical activity participation

The study results show that the mediating effect of self-efficacy accounts for 59.6% of the total effect, while the direct effect accounts for 40.4%. The mediating effect of self-efficacy in the relationship between social support and college students’ participation in physical activity is significant (0.261**). This finding indicates that social support not only directly influences physical activity participation but also indirectly promotes it by enhancing self-efficacy. This result aligns with numerous previous studies (Lin et al., 2024). Among the Chinese college student population, the mediating effect of self-efficacy is particularly significant among female students. This may be attributed to their lower participation levels in physical activities compared to male students, the strong influence of physical and psychological factors on self-efficacy, and the significant role of perceived school support in social support. In contrast, the mediating effect is less pronounced among male students. Nevertheless, even though the effect is less significant, improving self-efficacy can still enhance their physical activity participation levels. Research has shown that self-efficacy is one of the most reliable factors in physical activity, and there is a close relationship between adolescents’ physical activity and their self-efficacy in sports (Gao et al., 2021). Self-efficacy has been found to play a positive role, especially for female students, as it can indirectly encourage more active participation in physical activities by facilitating progress in behavior change related to physical activity (Ouyang et al., 2020).

This highlights the importance of developing Chinese college students’ self-efficacy in physical education, especially through supportive environments, positive reinforcement, and realistic goal setting. For female students in particular, establishing strong social support networks, providing psychological resources, and reducing barriers to participation can be especially beneficial. Ultimately, enhancing both social support and self-efficacy contributes not only to physical activity participation but also to broader physical and mental well-being.

## 5 Conclusion

The study identifies significant gender differences in physical activity participation among college students, with males (2.9 ± 0.78) showing higher levels than females (2.68 ± 0.653). For female students, social support enhances participation through the mediating role of self-efficacy. Thus, educational institutions should prioritize strategies that build self-efficacy—such as peer encouragement and positive role models—to motivate female students. Male students, who prefer competitive sports, also benefit from strong self-efficacy to manage competitive pressure and societal expectations. Institutions should offer appropriately challenging activities while supporting psychological resilience. In sum, gender-specific approaches are essential: enhancing self-efficacy and support for females and combining competitive opportunities with mental preparation for males. Such targeted efforts can effectively boost participation and foster a healthier campus culture.

## 6 Limitations and future directions

Although this study revealed that self-efficacy mediates the relationship between social support and physical activity participation among college students, this mediating effect was only significant among female students. The analysis for male students was less conclusive, indicating potential gender differences in the psychological mechanisms underlying physical activity behavior.

Several other limitations should also be acknowledged. First, the cross-sectional design limits the ability to infer causal relationships between social support, self-efficacy, and physical activity participation. Future research employing longitudinal or experimental designs is necessary to validate the temporal sequence and causal pathways. Second, reliance on self-reported data may introduce recall bias or social desirability effects, potentially affecting measurement accuracy. Incorporating objective measures—such as wearable fitness trackers or third-party assessments—could enhance data validity. Third, the study sample was drawn from a single private university, which may limit the generalizability of the findings to students from other types of institutions or regions. Expanding the sample to include public universities and students from different socioeconomic or cultural backgrounds would improve external validity.

Future research should also consider disaggregating analyses by gender, academic year, and field of study to better understand subgroup differences. Additionally, qualitative methods—such as interviews or focus groups—may provide richer insights into students’ perceptions of social support and self-efficacy in the context of physical activity. Identifying specific support mechanisms and motivational barriers across diverse student populations will help inform more tailored and effective intervention strategies.

## Data Availability

The raw data supporting the conclusions of this article will be made available by the authors, without undue reservation.
